# Accessibility of published research to practicing veterinarians

**DOI:** 10.5195/jmla.2018.196

**Published:** 2018-07-01

**Authors:** Jessica R. Page

**Affiliations:** Assistant Professor and Head, Hodesson Veterinary Medicine Library, University Libraries, The Ohio State University, 225 Veterinary Medicine Academic Building, Columbus, OH 43210

## Abstract

**Objectives:**

This study established the percentage of veterinary research articles that are freely available online, availability differences inside and outside of core veterinary medicine publications, sources and trends in article availability over time, and author archiving policies of veterinary journals. This research is particularly important for unaffiliated practitioners who lack broad subscription access and the librarians who assist them.

**Methods:**

Web of Science citation data were collected for articles published from 2000–2014 by authors from twenty-eight accredited US colleges of veterinary medicine. A sample of these articles was searched by title in Google Scholar to determine which were freely available online and their sources. Journals represented in this dataset and a basic list of veterinary serials were cross-referenced with the Sherpa/RoMEO database to determine author archiving policies and the percentage of articles that could potentially be made freely available.

**Results:**

Over half (62%) of the sample articles were freely available online, most of which (57%) were available from publishers’ websites. Articles published more recently were more likely to be freely available. More articles were found to be available in 2017 (62%) than in 2015 (57%). Most (62%) of the included journals had policies allowing authors to archive copies of their articles.

**Conclusions:**

Many articles are freely available online, but opportunity exists to archive additional articles while complying with existing copyright agreements. Articles in veterinary medicine–specific journals are less likely to be freely available than those in interdisciplinary journals. Requirements for federally funded research have likely influenced article availability and may continue to do so.

## INTRODUCTION

Access to freely available veterinary literature is of particular concern to librarians and specifically academic veterinary medical librarians, because they are frequently asked about access to the published literature by veterinary professionals who lack subscription access via institutional affiliation. These unaffiliated practitioners constitute the majority of veterinarians, fewer than 6% of whom work at colleges or universities [1].

Veterinary practitioners are increasingly making use of the published literature as part of their problem-solving and clinical decision-making processes, with a move toward online resources over time. A 1991 survey of veterinarians in the United States found that 78% of respondents had used the veterinary literature as part of their information-gathering process within the previous year, relying primarily on books that they owned [2]. A survey in 2000 of veterinarians in the United Kingdom again found that a majority sought information in books and journals that they owned or subscribed to themselves [3]. By 2010, the volume of veterinary information on the Internet had grown tremendously, and free search tools such as PubMed, Agricola, and Google Scholar made current scholarly literature increasingly discoverable to veterinary practitioners everywhere [4]. The improved discoverability of online content led to an increase in the use of journal articles in veterinary decision-making, with veterinarians indicating in 2011 that they read articles from a wide range of journals [5].

This broad use of the published literature by practicing veterinarians may reflect increasing emphasis on evidence-based veterinary medicine (EBVM), “the use of best relevant evidence in conjunction with clinical expertise to make the best possible decision about a veterinary patient,” in veterinary professional education [6]. More than half of the US veterinary librarians surveyed in 2011 provided some instruction in EBVM as part of a doctor of veterinary medicine (DVM) curriculum [7], and a 2016 survey of US and Canadian DVM curricula found similar rates of EBVM instruction [8]. The recently revised accreditation standards provided by the American Veterinary Medical Association Council on Education, which state that students must demonstrate specific competencies during their professional education including “critical analysis of new information and research findings relevant to veterinary medicine” [9], has led colleges of veterinary medicine to revise their DVM curricula to integrate EBVM instruction. While a 2012 review of the literature found that adoption of EBVM by practitioners was limited [10], these changes in the profession and professional instruction might have influenced the recent broad use of the literature by practitioners [5].

While academics in veterinary medicine increasingly emphasize the use of published literature in clinical decision-making through EBVM, it is not clear that this has had an impact on decisions related to publishing their own research. Despite a potential desire to make their research available to colleagues outside academia, veterinary authors might not be familiar with the nature and extent of open access (OA) publishing and/or archiving options that are available to them [11]. A small percentage of articles by veterinary authors are published in gold OA journals, defined by Suber as journals that make all articles OA from the time of publication [12]. In a study of articles published in 2006 and 2007 by authors from US and Canadian veterinary colleges, only 26% were freely available from PubMed Central or publishers’ websites, and the majority of those articles were not published in gold OA journals but were either freed after an embargo period or by an additional payment by the author [13]. This study also noted that the majority of gold OA publications were interdisciplinary rather than specific to veterinary medicine. A more recent study of publications in fields advocating for integration of scholarship in human, animal, and environmental health, known as One Health, found that only 29% of articles related to animal health topics were OA [14].

In light of the desire of veterinary practitioners to inform their decision-making using the published literature and of veterinary authors to make their work accessible to these practitioners, this paper addresses the following questions: First, how much of the veterinary literature is available for free online to anyone, and where is that literature found? Second, what are veterinary authors’ options for providing access to their research through choosing an OA publication venue or retaining the right to self-archive?

## METHODS

### Defining open access

For the purposes of this study, gold OA publications are those in which the publisher makes all journal content open immediately upon publication, often relying on author fees to fund access [12]. Green OA publications are those in which the publisher charges for subscription access, but authors retain the right to archive their work and make it public, subject to restrictions [12]. Sherpa/RoMEO, a database of publisher copyright and self-archiving policies owned by Sherpa Services, further differentiates among OA publications based on the version of a manuscript that an author may archive. As defined by Sherpa/RoMEO: green policies allow authors to immediately archive the pre-print as well as the post-print and/or publisher’s final version of an article; blue policies allow authors to archive the post-print or publisher’s version; yellow policies allow authors to archive the pre-print only; and white policies indicate no formal support for author archiving [15].

### Determining archiving policies of journals in which veterinary authors publish

Sherpa Services allows anyone to download the content of the Sherpa/RoMEO database as an extensible markup language (XML) file using their application programmer’s interface (API) [16]. For this study, the file was downloaded in February 2015. The third edition of the “Basic List of Veterinary Medical Serials” (n=123 journal titles; hereinafter “basic list”) was used to identify a limited list of journals specific to veterinary medicine [17]. Titles in the Sherpa/RoMEO database that occurred on the basic list were identified in Excel using title and international standard serial number (ISSN) in order to determine their Sherpa/RoMEO policies.

To gain a broader view of the OA policies of additional journals in which veterinary authors publish, the methodology from an earlier study was used to collect data from Web of Science (WOS) for articles published by authors from the 28 accredited US veterinary colleges from 2000–2014 [18]. The dataset of veterinary authors’ articles from WOS included 57,245 articles published over a 15-year period. The list of articles with their citation information was exported from WOS and then processed in Excel to remove duplicate articles and normalize journal titles. Document types were limited to article or review. Journals in this list (n=3,295 journal titles; hereinafter “WOS journals”) were matched with their Sherpa/RoMEO policies by title or ISSN using the same method as for titles on the basic list. A total of 118 of the 123 titles on the basic list were included among the 3,295 WOS journals.

### Determining article availability

A sample of these articles was used to determine the availability of free, online copies of articles. The sample size for each year was calculated to provide sufficient power to compare availability across years (confidence interval=0.05, confidence level=0.95, Z score=1.96, standard deviation=0.5) for an average sample size of 348 articles per year and 4,871 total articles. Change in availability over time was analyzed using time series regression.

To select articles for the sample, the articles were sorted into separate lists for each publication year. Excel’s random number generator was used to assign a random number to each article in the year’s list, and then the list was sorted by random number. The sample was selected by choosing the first *n* articles listed in this random order for each year.

The full title of each article was copied and pasted to a Google Scholar search. Google Scholar is a free search tool from Google that indexes scholarly articles from many sources on the Internet [19]. Searches were conducted from an off-campus location using no university proxy service or virtual private network (VPN) so that only full-text content that was available to all users, rather than those affiliated with a university, would be identified. The presence or absence of a link to the full text of the article from Google Scholar was documented. When a link for full-text access was present, the type of source to which Google Scholar directed the user was noted. Google Scholar groups multiple copies of a work into a single result and preferentially displays full-text links to (1) publisher’s websites; (2) government websites, including PubMed Central; (3) academic websites, including institutional repositories and departmental or faculty websites; (4) ResearchGate or Academia.edu; or (5) other sources. While there were often several full-text options available, for the purposes of this study the first full-text result displayed was used.

The searches were conducted August 2015 and repeated January 2017 to determine whether and how availability had changed in that interval.

## RESULTS

### Freely available articles

In August 2015, searching for veterinary articles by title in Google Scholar showed that 57% of the articles sampled were freely available to all users. In January 2017, 62% of these articles were freely available. Data from 2017 are used for all results below.

**Figure 1:**
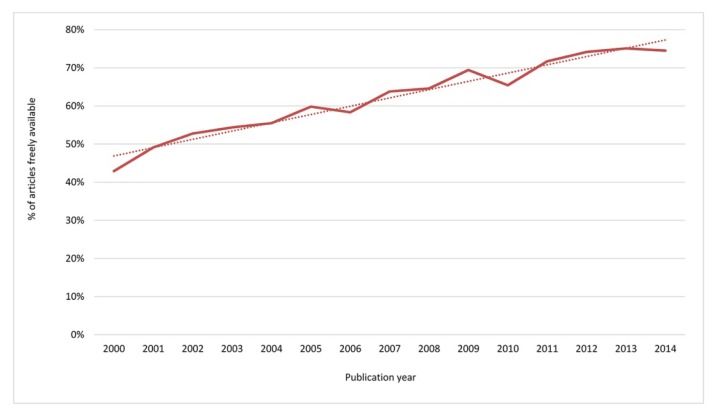
Trend in free, online article availability over the study period The dotted line shows the linear trend in availability over time.

Newer articles were more likely to be freely available than older articles, as shown by a trend of increasing availability of articles by publication year over the study period following a linear model (R2=95.8%; R2(adj)=95.5%; F(17222.9,61.4)=295.28; *p*<0.0001). Availability peaked (75%) for articles published in 2013, and slightly fewer articles published in 2014 were available, possibly due to embargo periods at publishers’ websites (Figure 1).

**Figure 2:**
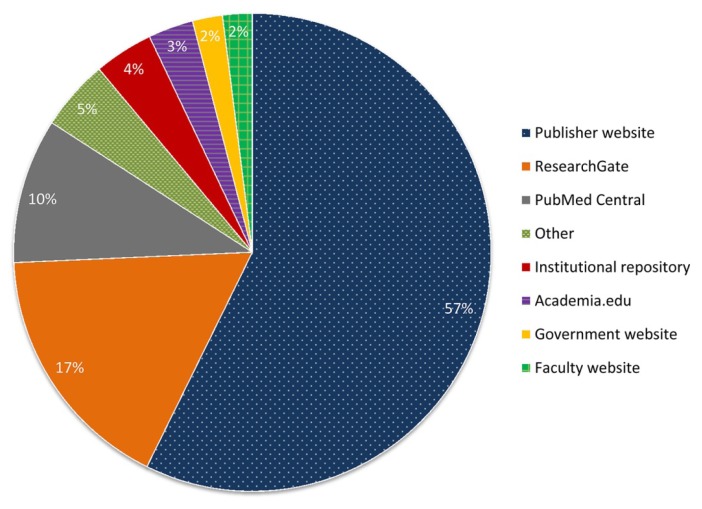
Sources of freely available articles

Based on the way Google Scholar presents results, the majority of freely available articles were available from the publisher’s website (Figure 2). The second most common source was ResearchGate (17%), followed by PubMed Central (10%). Less common were institutional repositories, personal faculty websites, government websites, and other sources.

**Figure 3:**
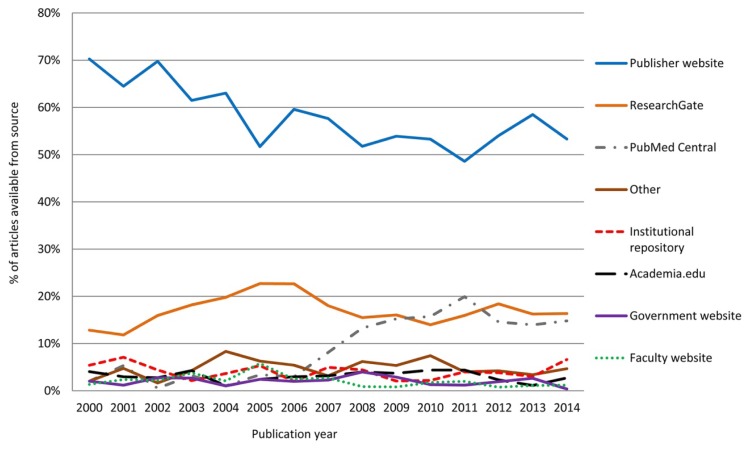
Sources of freely available articles over time

The sources of freely available articles varied by article publication date (Figure 3). Articles published more recently were less likely to be found on publishers’ websites than those published earlier in the study period, possibly reflecting publishers’ embargo periods or embargo periods permitted by funding agencies. Articles available from PubMed Central increased over the study period, during which PubMed Central increased the catalog of journals for which it archived some or all content, in part due to funding agency requirements. During the summer of 2016, Google Scholar resumed indexing content from Academia.edu, a practice it had previously performed and then ceased. However, Google Scholar displayed results from Academia.edu for only 3% of the total available articles.

Articles published in core veterinary medicine journals were less likely to be freely available online than articles that were published more broadly. Across all publication years (2000–2014), only 47% of articles in journals on the basic list were freely available, while 75% of articles that were published in WOS journals but were not in the basic list were freely available. Journals from which most or all articles were found to be freely available were generally not focused on veterinary medicine. There were 630 WOS journals for which at least 95% of the articles searched were freely available; of these, only 21 titles (3%) were on the basic list.

### Archiving policies

Many journals in which veterinary authors published allowed self-archiving as indicated by Sherpa/RoMEO. Sherpa/RoMEO listed policies for 68% of the journals in WOS with veterinary publications, while 32% of these journals either did not have a policy on self-archiving or did not have a policy included in the Sherpa/RoMEO database.

**Figure 4:**
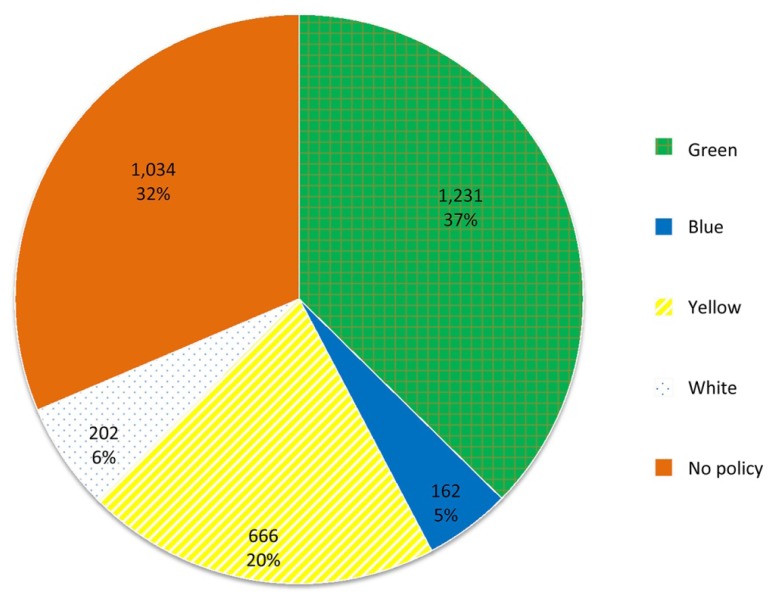
Sherpa/RoMEO archiving policies of Web of Science journals with publications by college of veterinary medicine authors

Over half (62%) of the journals in WOS with veterinary publications had policies that permit author self-archiving (green, blue, or yellow), whereas 6% did not formally support author archiving (white) (Figure 4). The WOS journals, which included journals outside of veterinary medicine, and the veterinary medicine–only basic list had similar percentages for each Sherpa/RoMEO policy category. The journals that veterinary authors published in most frequently were more likely to permit archiving: 80% of the top 25 WOS journals had Sherpa/RoMEO archiving policies that permitted author archiving.

There was no clear relationship between Sherpa/RoMEO author archiving policies and publisher type. All Sherpa/RoMEO archiving policy categories were represented among commercial and society publishers. Journals without policies listed in the Sherpa/RoMEO database included gold OA publishers, individual journals not covered by their commercial publisher’s broader policy, and society publications.

## DISCUSSION

This study found that a majority of articles published by authors at US veterinary colleges were freely available online and that online availability was increasing over time. Most of the available articles could be found on publishers’ websites, and the remainder were hosted on a mix of platforms representing forms of article sharing that fell within author copyright agreements (e.g., institutional or government repositories) and those that likely did not (e.g., ResearchGate or Academia.edu). Most of the articles that were freely available were not from the journals that publish material most relevant to the clinical practitioners who would rely on free access. The prevalence of journals that permitted at least some form of article sharing by authors suggested that more articles could be made freely available.

### Archiving policies

While a majority of the journals with Sherpa/RoMEO policies allowed author self-archiving, only 4% of the available articles found via Google Scholar were author-archived copies. In part, this would be due to Google Scholar preferentially linking to publishers’ full-text copies (57%), even when an author-archived copy might be available. However, 27% of available articles were from sources other than either publishers or publisher-approved repositories (e.g., ResearchGate, Academia.edu, personal websites, and other websites).

Because the Sherpa/RoMEO policies used for this study were from a snapshot in time, whereas article publication and archiving took place over a fifteen-year period, it is difficult to tell what policies might have applied to an article at a given time. This was especially challenging given the changes to the publishing landscape that took place during the study period. This included the National Institutes of Health requirement that authors who received funding deposit their articles in PubMed Central upon acceptance, a policy that the agency enacted in 2009 [20] and began enforcing more strictly in 2013 [21], and the more wide-reaching memo from the White House Office of Science and Technology Policy in 2013 requiring that nearly all federally funded research be made freely available [22]. The White House memo led to public access policies from other funding agencies that are relevant to veterinary medicine, including the US Department of Agriculture [23] and the National Science Foundation [24]. These initiatives resulted in publishers changing the permissions that they grant to authors and their publication practices, including making the published version of federally funded articles openly available on their own websites, where they would receive priority linking and traffic from Google Scholar searches. These changes might, in part, explain the increasing number of open articles available over the study period, particularly from publishers and PubMed Central.

### Trends in article availability

Journals with restrictive author archiving privileges and closed-access back files were, as expected, less likely to be freely available online but were not completely unavailable. Copies were often posted by authors, interested organizations, or others on websites of varying degrees of repute. The presence of these articles online suggested demand for them and indicated that publishers who did not make content freely available on their own websites might be losing page views that they would otherwise receive. Publishers might implement restrictive archiving policies intending to protect their copyright and a potential revenue source and to ensure that the official, published copy of an article is the version that is used. However, the presence of “unofficial” copies of articles in places such as ResearchGate, Academia.edu, or personal author websites indicates that restrictive archiving policies do not necessarily prevent content from being posted online. While outside the scope of this study, it is worth noting that academics also share articles that are not freely available online via sources that are not indexed by Google Scholar, such as SciHub [25], Twitter (#icanhazpdf), Reddit, and email [26, 27].

Because articles in journals that narrowly cover veterinary medicine (i.e., the basic list) were less likely to be freely available online than those in auxiliary or interdisciplinary fields (i.e., the remainder of the WOS journals), much of the core of the veterinary literature was unavailable to most veterinarians. As a result, much of the literature that is of clinical importance to veterinary practitioners is less likely to be available to them than basic research. Publishers of veterinary medicine journals have an opportunity to revisit their publishing models and might consider following in the path of the high-impact *Journal of Veterinary Internal Medicine,* which changed in 2014 from traditional, restricted subscription access to full OA for all issues.

Given the relatively low number of gold OA journals in veterinary medicine, it was surprising to find that the majority of freely available, online content was at publishers’ websites. A number of factors might contribute to this.

One factor was that veterinary authors publish in interdisciplinary gold OA journals such as PLOS ONE. The number of articles published in PLOS ONE increased over the study period, and by 2013, PLOS ONE was the top interdisciplinary journal in which veterinary authors published. Another major factor was publishers uploading journal back files with OA after an embargo period. For example, the American Society for Microbiology (ASM) publishes a number of journals that are highly represented in this study (e.g., *Journal of Virology, Infection and Immunity,* and *Journal of Clinical Microbiology,* all of which are in the top twenty-five journals searched). The articles are freely available at the ASM website after six months or one year in the case of review articles [28]. Articles in the *Proceedings of the National Academy of Sciences* are also made freely available at their website after six months [29].

In other cases, authors may pay publishers to make a specific article OA immediately upon publication in a journal that is otherwise available only to subscribers (e.g., Wiley’s OpenOnline option). This study did not determine whether or how often this was the case. For the journals that open their content after an embargo period, it would be difficult or impossible to tell after that period if an author paid for immediate open access.

A combination of funding agency requirements, publishers’ policies, publication in interdisciplinary OA journals, and self-archiving by authors who wish to share their work has resulted in a large and increasing number of articles in the veterinary medical literature being freely available online. It will be interesting to see whether these trends of increasing access to both new and older veterinary medical literature continue. Given political changes, it is possible that changes will occur to the federal funding mandates that require publishers to make federally funded research open. Publishers may return to earlier restrictions for new articles or back files, or in light of the momentum in veterinary medicine and other disciplines toward improving OA to the literature, they may choose to continue making the work that they publish available. The latter seems to be happening in at least some cases. For instance, as of January 2017, the Bill & Melinda Gates Foundation is partnering with the American Association for the Advancement of Science (AAAS) to increase open access to foundation-funded research [30]. Beginning in February 2017, the Electrochemical Society is working to make all of its publications and data OA [31]. Given veterinary authors’ interest in providing scientific evidence to veterinary, public health, and other practitioners, authors will likely continue to fill gaps in availability by archiving their own work, whether in institutional repositories, on commercial sites such as ResearchGate, or elsewhere.

A majority of articles in veterinary medicine are freely accessible online to all users, and that accessibility is increasing over time. Publishers contribute to the accessibility of articles both through author archiving policies and OA materials on their own platforms. Librarians can work with authors on issues regarding OA publishing options, copyright retention, and author archiving permissions. Librarians can also direct unaffiliated patrons to tools such as Google Scholar or the Unpaywall browser plugin [32] to help them easily connect with free, legal copies of the published evidence they need in their practice.

While this study focuses on veterinary medicine, the interdisciplinary nature of work published by veterinary authors suggests implications beyond this field. Veterinary medicine is an integral part of One Health, the idea that scholarship in human, animal, and environmental health should be integrated and collaborative. Trends seen in veterinary publication reflect interdisciplinary research and can be seen as a case study for broader biomedical scholarship.

### Limitations of the study

The articles examined in this study were limited to those indexed in WOS. WOS does not index all articles published by veterinary authors, nor does it index all veterinary journals, notably omitting several titles from the basic list of veterinary serials. Also, articles included in this study were limited to those published by authors affiliated with colleges of veterinary medicine. This excludes works written by authors from relevant programs, such as veterinary science or veterinary technology, that are not part of colleges of veterinary medicine. It also excludes articles with only nonacademic authors, including those working in private practice, government agencies, or pharmaceutical or pet food companies.

An additional limitation is presented by using Google Scholar to locate available articles. Articles may be freely available from more than one source, but Google Scholar displays just one on its initial results screen. Google Scholar’s algorithm preferentially displays freely available full-text copies of articles from publishers’ websites first, followed by government and academic sources including institutional repositories, and then by ResearchGate, Academia.edu, and other sources. Any additional sources of articles, which may include more full-text options, are masked behind Google Scholar’s preferred source, though users can view them by selecting “All [number] versions” from the results page. For example, a given search can locate and link to an article at the publishers’ website, but that same article may also be available from PubMed Central and an institutional repository. This study only documented the first source of freely available full text displayed rather than all versions.

## References

[1] American Veterinary Medical Association Market research statistics: U.S. veterinarians 2016 [Internet].

[2] Pelzer NL, Leysen JM (1991). Use of information resources by veterinary practitioners. Bull Med Libr Assoc.

[3] Wales T (2000). Practice makes perfect? vets’ information seeking behaviour and information use explored. Aslib Proc.

[4] Larson RL (2010). Access to scientific literature in rural veterinary practice. Online J Rural Res Policy.

[5] Huntley SJ, Dean RS, Massey A, Brennan ML (2016). International evidence-based medicine survey of the veterinary profession: information sources used by veterinarians. PLOS ONE.

[6] University of Nottingham Evidence-based veterinary medicine (EVM) [Internet].

[7] Dinkelman AL, Viera AR, Bickett-Weddle DA (2011). The role of veterinary medical librarians in teaching information literacy. J Vet Med Educ.

[8] Shurtz S, Fajt V, Heyns EP, Norton HF, Weingart S (2017). Teaching evidence-based veterinary medicine in the US and Canada. J Vet Med Educ.

[9] American Veterinary Medical Association (2017). Council on Education (COE) accreditation policies and procedures: requirements [Internet].

[10] Vandeweerd JM, Kirschvink N, Clegg P, Vandenput S, Gustin P, Saegerman C (2012). Is evidence-based medicine so evident in veterinary research and practice? history, obstacles and perspectives. Vet J.

[11] Christopher MM, Young KM (2015). Awareness of “predatory” open-access journals among prospective veterinary and medical authors attending scientific writing workshops. Front Vet Sci.

[12] Suber P (2015). Open access overview [Internet].

[13] Nault AJ (2011). Open access of publications by veterinary faculty in the United States and Canada. J Vet Med Educ.

[14] Vreeland CE, Alpi KM, Pike CA, Whitman EE, Kennedy-Stoskopf S (2016). Access to human, animal, and environmental journals is still limited for the One Health community. J Med Libr Assoc.

[15] Definitions and terms [Internet].

[16] (2013). Application programmers’ interface [Internet].

[17] Ugaz AG, Boyd CT, Croft VF, Carrigan EE, Anderson KM (2010). Basic list of veterinary medical serials, third edition: using a decision matrix to update the core list of veterinary journals. J Med Libr Assoc.

[18] Page JR, Moberly HK, Youngen GK, Hamel BJ (2014). Exploring the veterinary literature: a bibliometric methodology for identifying interdisciplinary and collaborative publications. Coll Res Libr.

[19] Inclusion guidelines for webmasters [Internet].

[20] National Institutes of Health (2016). NIH public access policy details [Internet].

[21] Matthews S (2013). NIH will withhold grant money to enforce public-access policy. Nat Med.

[22] Stebbins M (2013). Expanding access to the results of federally funded scientific research [Internet].

[23] United States Department of Agriculture (2014). Implementation plan to increase public access to results of USDA-funded scientific research [Internet].

[24] National Science Foundation (2015). Public access plan: today’s data, tomorrow’s discoveries: increasing access to the results of research funded by the National Science Foundation [Internet].

[25] Greshake B (2016). The SciHub data part 2: academic pirates [Internet].

[26] Gardner C, Gardner G Bypassing interlibrary loan via Twitter: an exploration of# icanhazpdf requests [Internet]. http://eprints.rclis.org/24847/.

[27] Gardner CC, Gardner GJ (2017). Fast and furious (at publishers): the motivations behind crowdsourced research sharing. Coll Res Libr.

[28] American Society for Microbiology Frequently asked questions about institutional subscriptions [Internet].

[29] Subscriptions FAQ [Internet]. Proceedings of the National Academy of Sciences.

[30] AAAS and Gates Foundation partnership announcement [Internet].

[31] Electrochemical Society A new model for scientific publishing [Internet].

[32] Unpaywall [Internet].

